# Supplementary material for: A whole-animal phenotypic drug screen identifies suppressors of atherogenic lipoproteins
Source: eLife. 2026 Aug 27;14:RP105314. doi: 10.7554/eLife.105314 (PMC13521566; doi:10.7554/eLife.105314)
Supplement: Supplementary file 2 [file elife-105314-code1.zip › JHDL_primary_result/Cinnamon┬áoil/Cinnamon┬áoil_JHDL_29_027_057_2019_12_06_13_27.pdf]

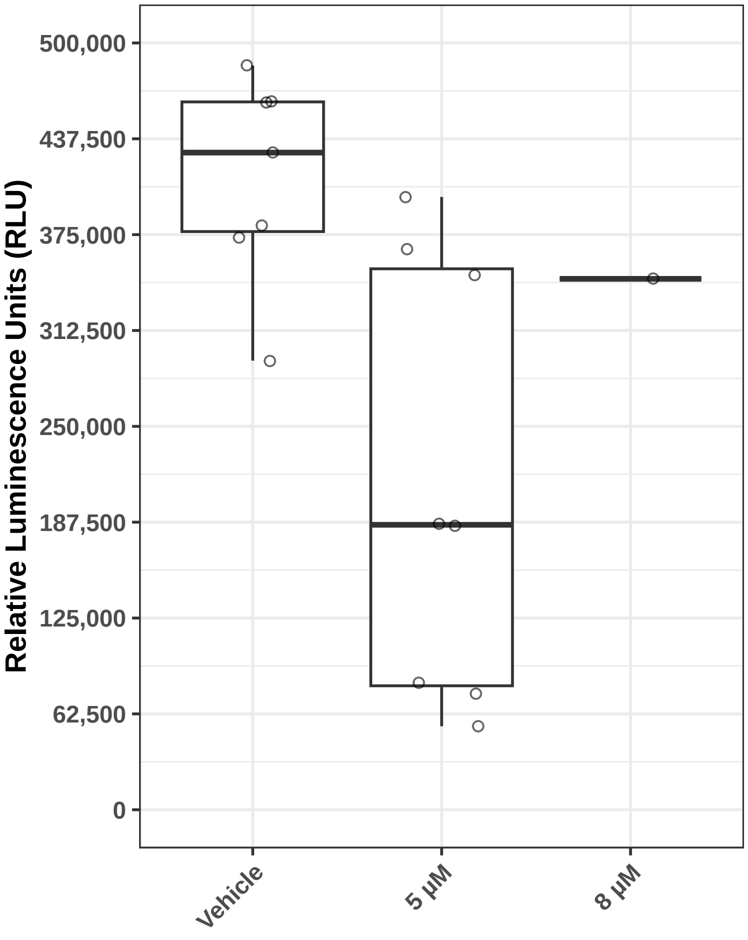

Relative Luminescence Units (RLU)
500,000
437,500
375,000
312,500
250,000
187,500
125,000
62,500
0
Vehicle
5 μM
8 μM
